# Left Internal Carotid Artery Dissection Secondary to Minor Trauma: A Case Report Involving “Headbanging” in a Heavy Metal Band

**DOI:** 10.1155/crvm/9589977

**Published:** 2026-10-27

**Authors:** Louissa Srama Rosner Cidral, Eduarda Nakoula, Kristel Back Merida, Ana Carolina Andrade, Luiz Penzo, Leonardo Gilmone Ruschel

**Affiliations:** ^1^ Neurology and Neurosurgery Department NeuroDOC Curitiba Brazil; ^2^ Neurology and Neurosurgery Department Hospital Santa Cruz Curitiba Paraná Brazil

**Keywords:** cervical artery dissection, headbanging, internal carotid artery dissection, ischemic stroke, minor trauma

## Abstract

**Introduction:**

Internal carotid artery dissection (ICAD) is an uncommon but clinically significant cause of ischemic stroke in young adults, accounting for approximately 10%–25% of cases in this population. It is frequently associated with minor trauma or repetitive cervical movements. Headbanging, characteristic of heavy metal music performances, involves vigorous, repetitive movements of the head and neck and may increase the risk of cervical vascular injury, including ICAD.

**Case Report:**

A 37‐year‐old male musician with no prior neurological disease presented with severe headache and transient focal neurological symptoms following heavy metal performances involving headbanging. On examination, blood pressure was 130/80 mmHg, and no focal neurological deficits were observed (NIHSS score: 0). Computed tomography angiography demonstrated a subocclusive thrombus involving the distal cervical and petrous segments of the left internal carotid artery, with distal hypoperfusion. Digital subtraction angiography subsequently confirmed a left ICAD of the petrous segment with approximately 80% luminal stenosis. Management included anticoagulation with dabigatran, strict blood pressure control, and multidisciplinary follow‐up, including psychological support. The patient was discharged with guidance to avoid abrupt cervical movements and to continue regular outpatient and imaging follow‐up.

**Discussion:**

Although uncommon, the association between headbanging and ICAD warrants consideration in young patients presenting with ischemic stroke in the absence of traditional cardiovascular risk factors. Repetitive, forceful cervical movements may cause arterial wall injury, predisposing to dissection. Early diagnosis is essential to guide appropriate management and prevent complications.

**Conclusion:**

This case highlights the importance of recognizing activities involving repetitive, vigorous cervical movements, such as headbanging, as potential etiological factors for arterial dissection in young adults. The absence of follow‐up imaging is a limitation of this case report; nonetheless, it remains useful in raising awareness that may facilitate earlier diagnosis and improve clinical outcomes.

## 1. Introduction

Internal carotid artery (ICA) dissection is a recognized cause of ischemic stroke in young adults and is often associated with minor or trivial trauma [1]. Despite its low overall incidence, cervical artery dissection accounts for a considerable proportion of strokes in this age group, an estimated 10%–25% of cases [2]. Mechanical triggers such as cervical manipulation, contact sports, and repetitive abrupt head movements have been implicated as precipitating factors and may contribute to underdiagnosis when they are not actively investigated [1].

Headbanging, a movement characteristic of heavy metal music, involves repetitive, rapid, and forceful flexion, extension, and rotation of the cervical spine. Although often perceived as harmless, this practice may expose cervical vascular structures to repetitive mechanical stress, increasing the risk of arterial wall injury. Reports linking headbanging to cervical artery dissection remain scarce, and awareness of this association is still limited [1–3].

We report the case of a 37‐year‐old heavy metal musician who developed a left internal carotid artery dissection (LICAD) following repetitive headbanging, highlighting the importance of considering minor trauma mechanisms in the etiological evaluation of ischemic stroke in young adults. Although cervical artery dissection following minor trauma has been previously described, reports specifically involving headbanging remain extremely uncommon.

This case report contributes to the limited literature by describing the clinical presentation, vascular imaging findings, management strategy, and follow‐up of a LICAD involving the petrous segment following repetitive, vigorous cervical movements. Greater awareness of this potential association may support earlier diagnosis in young patients without traditional vascular risk factors.

## 2. Methodology

This is a descriptive, retrospective, and qualitative case report based on the analysis of clinical data from a 37‐year‐old patient diagnosed with LICAD following possible minor trauma sustained during headbanging while performing in a heavy metal band. Data were obtained from the patient′s electronic medical records, covering the hospitalization period between September and October 2024, as well as outpatient follow‐up visits after discharge.

Data collected included a detailed clinical history, encompassing relevant comorbidities such as systemic arterial hypertension and prior ablation for Wolff–Parkinson–White syndrome; imaging findings (cranial computed tomography angiography and cerebral arteriography); and neurological examination findings, including the NIH Stroke Scale (NIHSS) score, a description of the therapies instituted, and the clinical course during follow‐up. Imaging studies were reviewed with the support of the neuroradiology team, detailing the extent of the dissection and the presence of thrombus. The patient′s clinical course was discussed in light of the recent literature on ICA dissection associated with minor trauma.

This case report was conducted in accordance with the ethical principles of the Declaration of Helsinki. Written informed consent was obtained from the patient for the publication of this case report and the accompanying images. Patient confidentiality was maintained throughout the preparation of the manuscript.

## 3. Case Report

His medical history was notable for Wolff–Parkinson–White syndrome, for which he had undergone successful ablation of a left posteroinferior accessory pathway in April 2024, and systemic arterial hypertension treated with losartan 50 mg twice daily and amlodipine 5 mg twice daily. Anticoagulation with dabigatran (Pradaxa) 150 mg every 12 h was initiated, with pantoprazole 40 mg once daily prescribed for gastric protection. Since discharge, the patient had been performing his professional duties remotely.

On physical examination, blood pressure was 130/80 mmHg, muscle strength was preserved, and no focal neurological deficits were detected; the NIHSS score was 0, indicating no measurable neurological impairment.

Computed tomography angiography demonstrated findings suggestive of a subocclusive thrombus in the left ICA involving the distal cervical and intracranial petrous segments, with associated signs of distal hypoperfusion in the corresponding arterial territory. Cerebral angiography performed on September 18, 2024, confirmed a LICAD in the petrous segment measuring approximately 5.71 mm in length, with significant luminal stenosis estimated at approximately 80%, as seen in Figures 1, 2, 3, 4, 5, and 6.

**Figure 1:**
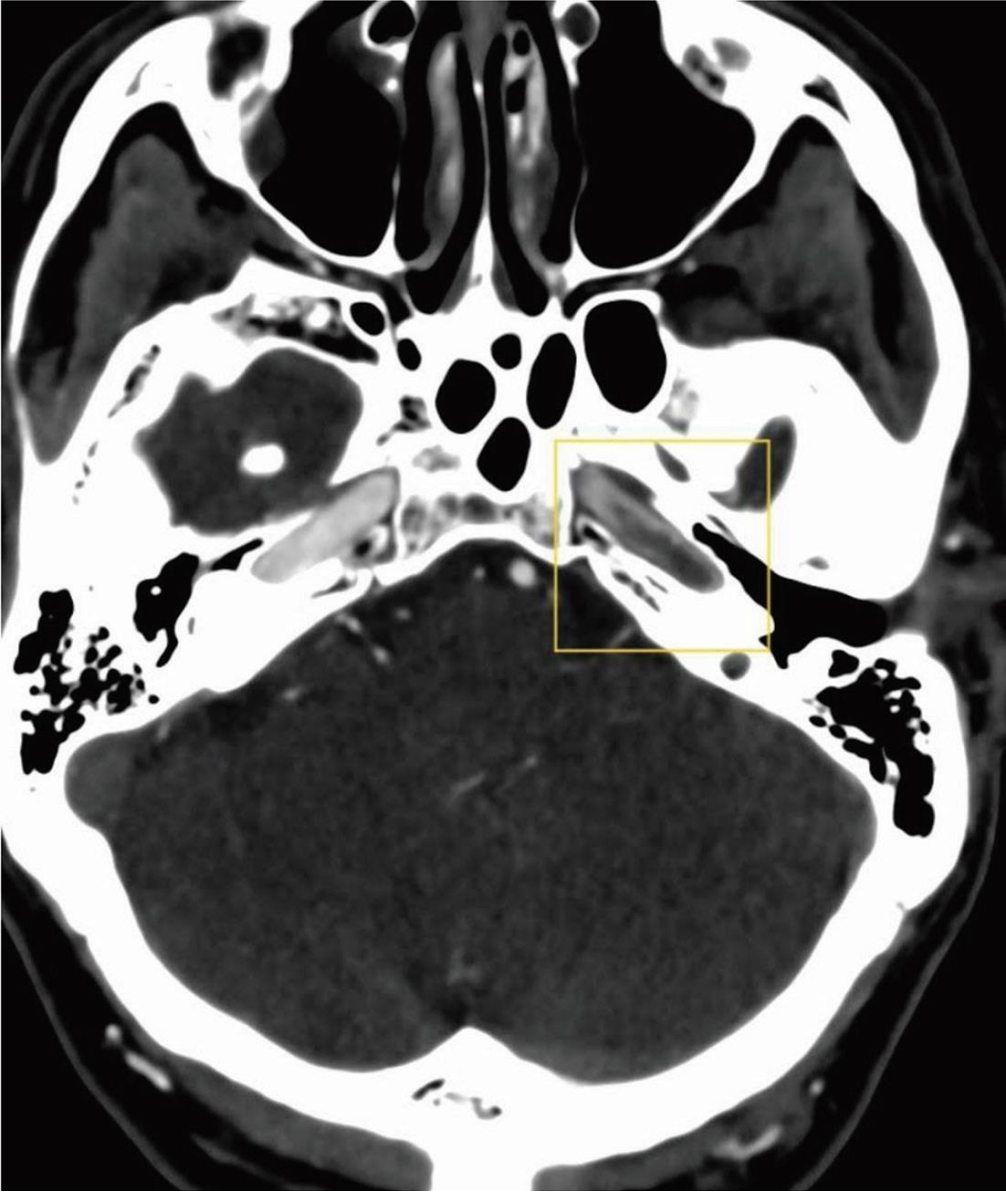
Cranial computed tomography with bone window. This image shows an axial cranial computed tomography (CT) scan focused on the skull base, with particular emphasis on the temporal bone region. The highlighted rectangle delineates the area of the internal auditory canal, where a structure suggestive of a space‐occupying lesion or evidence of bony alteration can be observed. The bone window setting allows detailed visualization of adjacent structures, including the mastoid air cells and the skull base. In addition, the image suggests the presence of a subocclusive thrombus in the left internal carotid artery (ICA), involving the distal cervical and intracranial petrous segments. There are also signs of distal hypoperfusion in the corresponding arterial territory, indicating compromised blood perfusion in the affected region. *Source:* Property of the authors.

**Figure 2:**
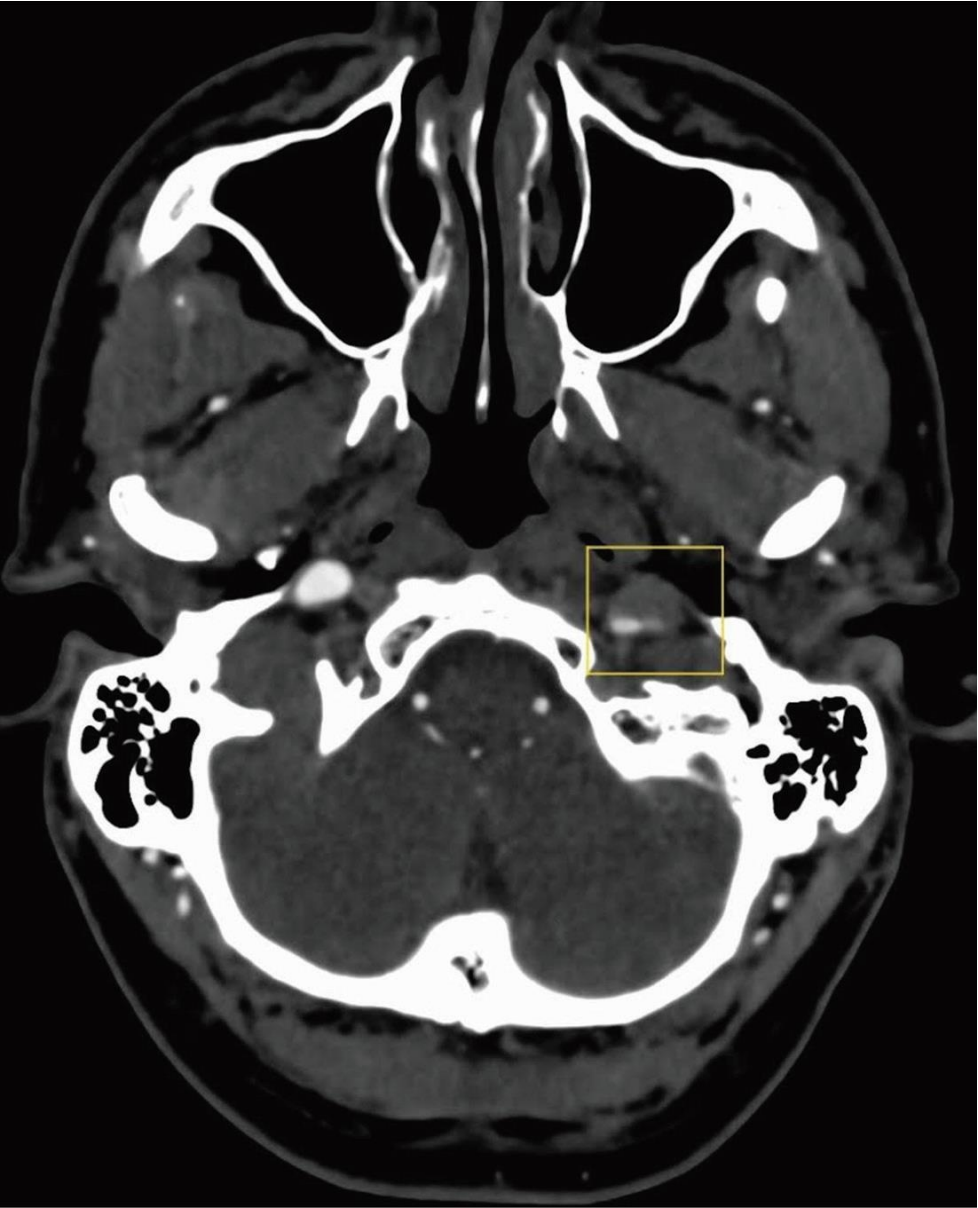
Contrast‐enhanced cranial tomography. This axial contrast‐enhanced cranial computed tomography (CT) scan depicts the posterior fossa and the surrounding vascular anatomy. The highlighted rectangle indicates an area of interest that may correspond to a vascular structure or a lesion demonstrating contrast enhancement. The image also suggests the presence of a subocclusive thrombus in the left internal carotid artery (ICA), involving the distal cervical and intracranial petrous segments. In addition, there are signs of distal hypoperfusion in the corresponding arterial territory, indicating impaired regional blood perfusion. *Source:* Property of the authors.

**Figure 3:**
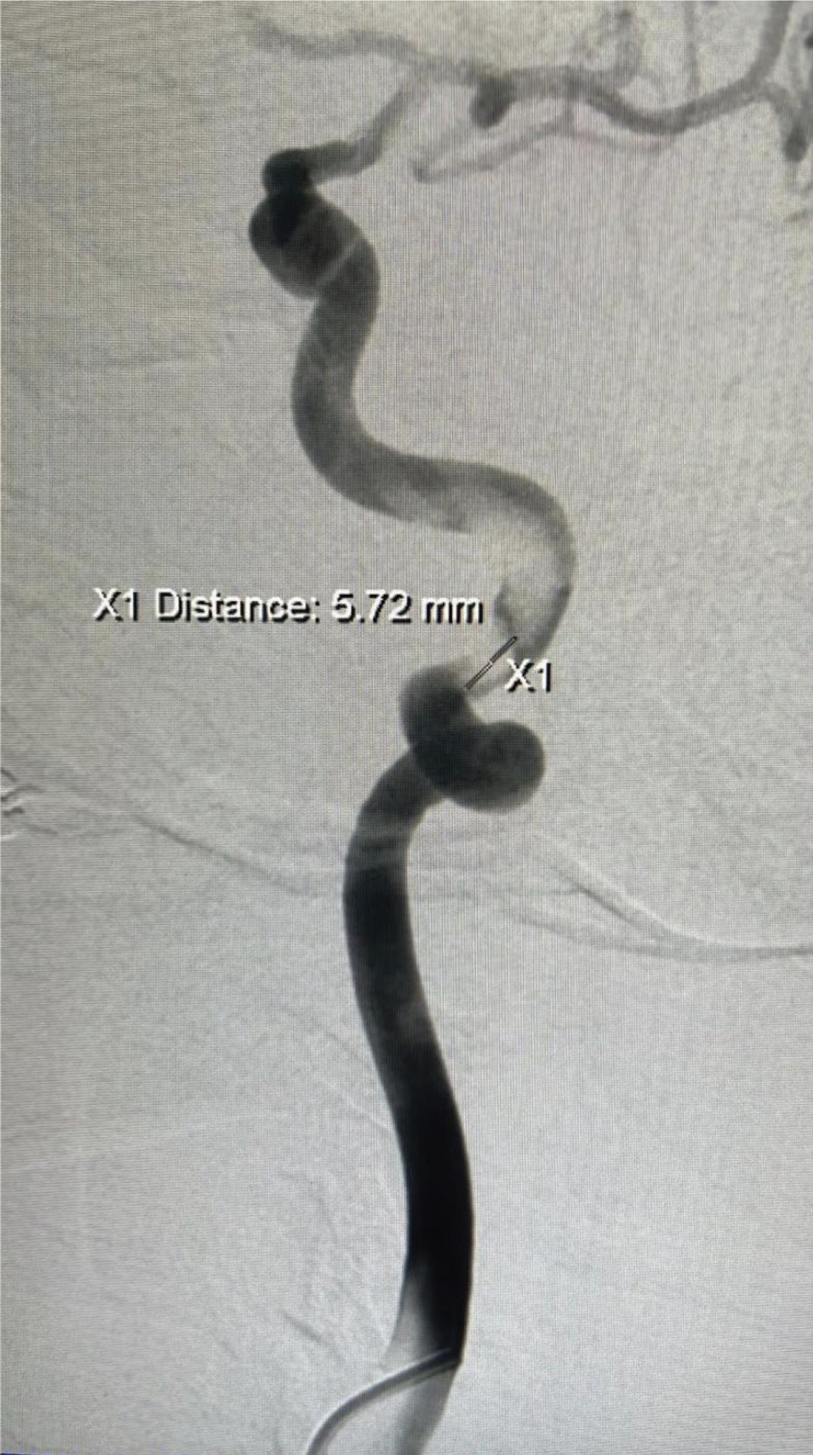
Cerebral angiography—integral. Digital subtraction angiography demonstrating the left internal carotid artery with abnormality involving the petrous segment and marked luminal narrowing. *Source:* Property of the authors.

**Figure 4:**
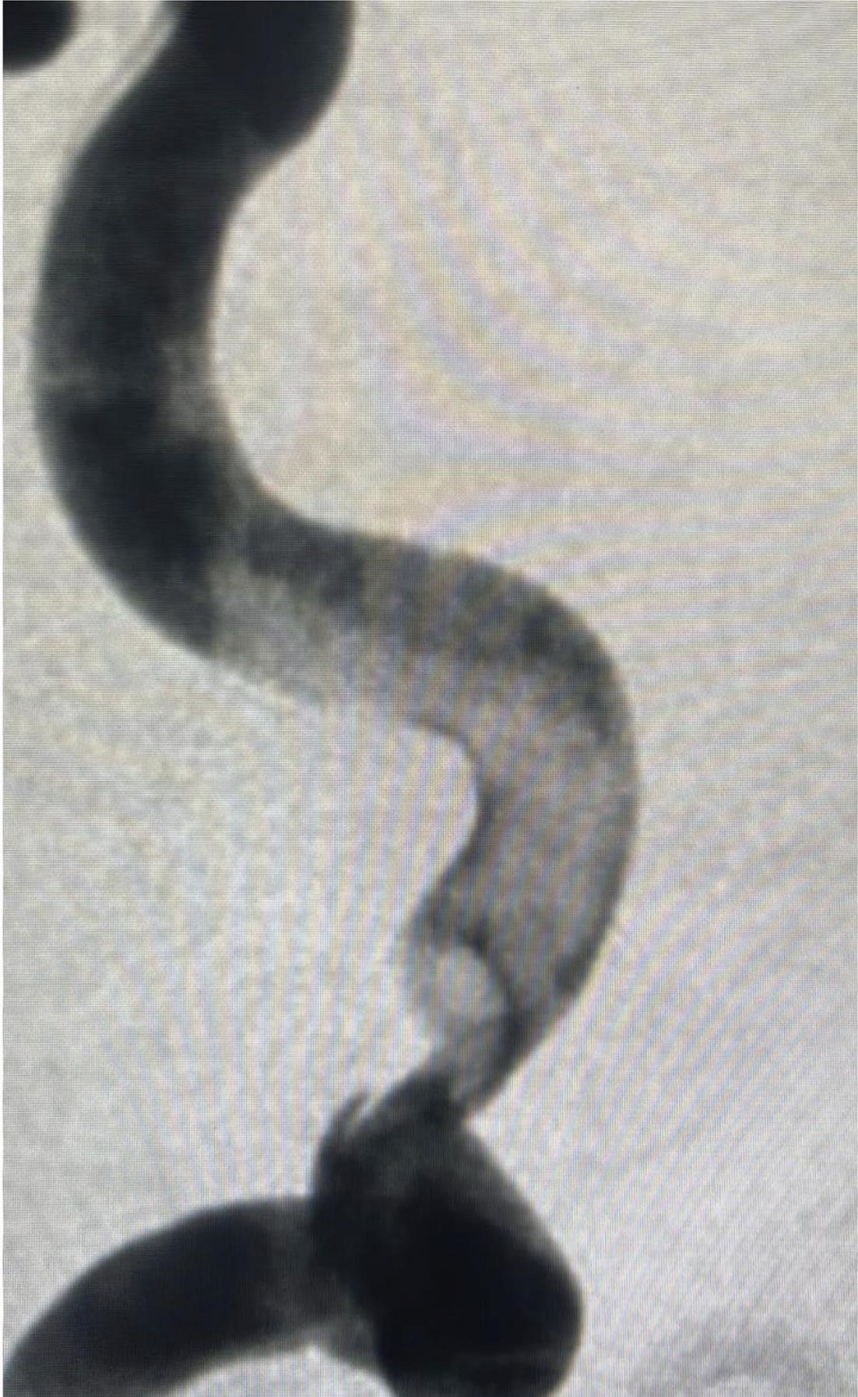
Cerebral angiography—zoom. Selective angiographic view demonstrating dissection of the petrous segment of the left internal carotid artery, measuring approximately 5.71 mm in length and associated with approximately 80% luminal stenosis. *Source:* Property of the authors.

**Figure 5:**
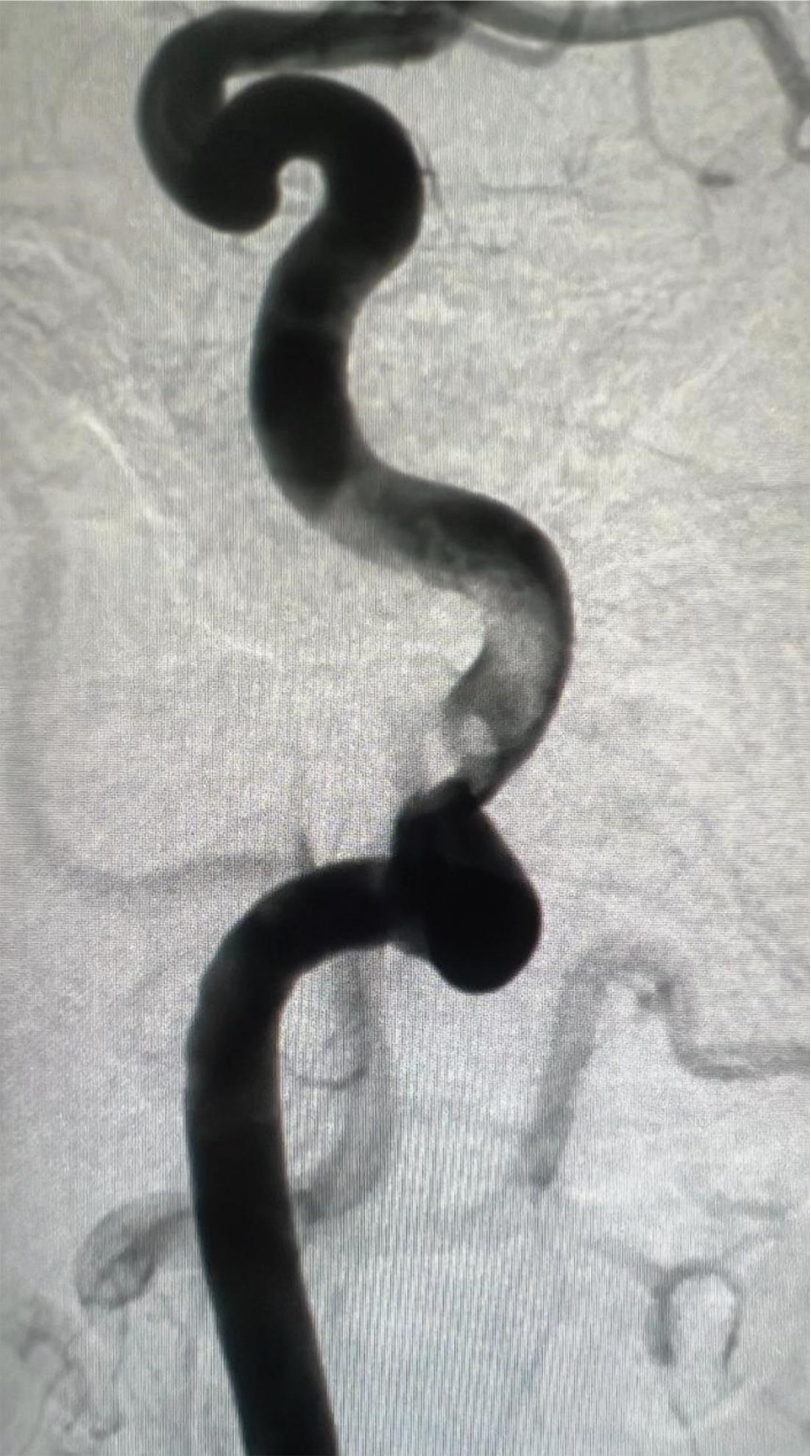
Cerebral angiography—internal carotid artery. Angiographic image demonstrating the left internal carotid artery and the abnormality involving the petrous segment, consistent with the documented arterial dissection. *Source:* Property of the authors.

**Figure 6:**
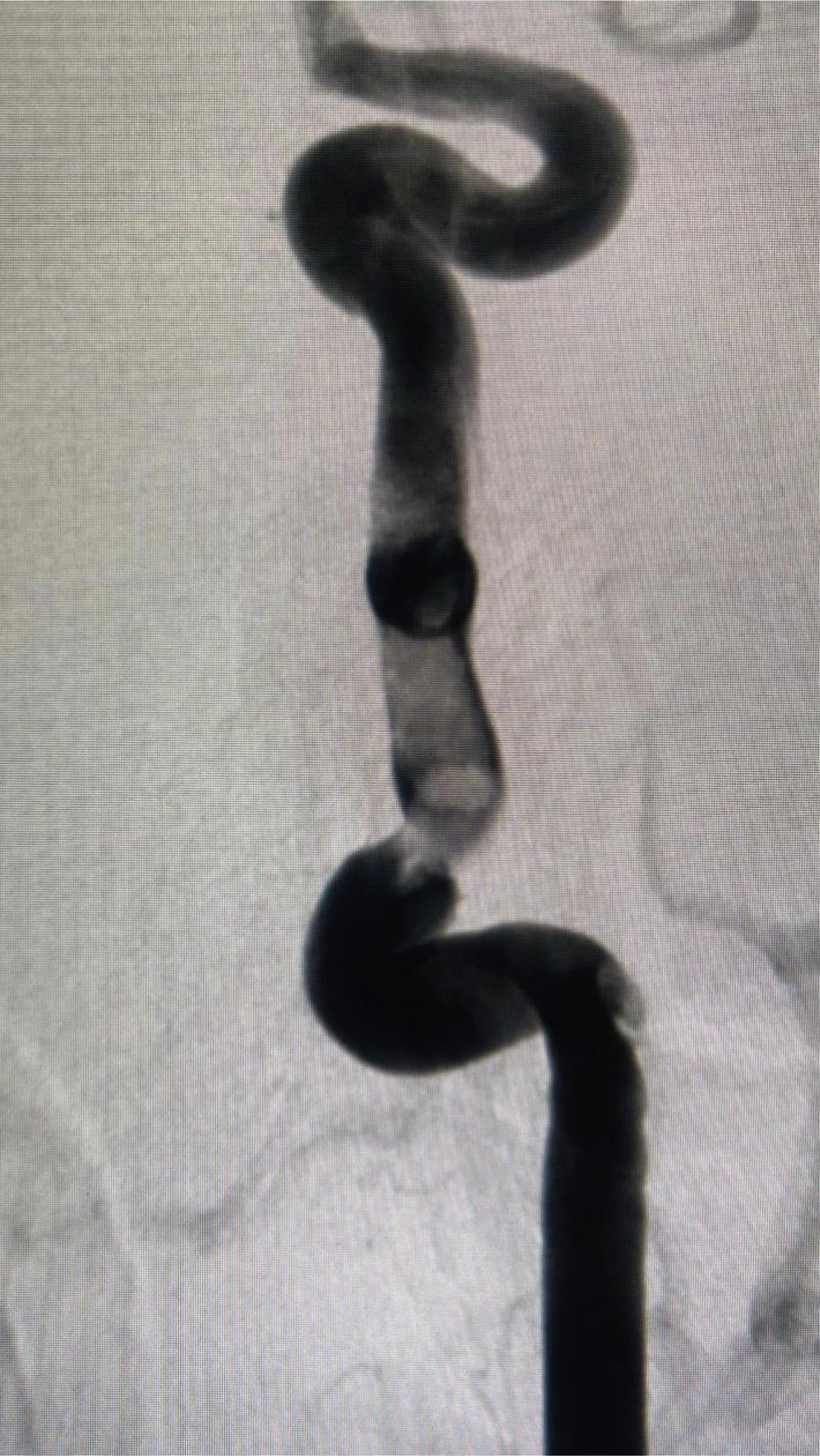
Cerebral angiography—internal carotid artery petrous segment. Additional angiographic projection demonstrating the dissected petrous segment of the left internal carotid artery with marked luminal narrowing. *Source:* Property of the authors.

Therapeutic management focused on strict blood pressure control and continuation of anticoagulation. Follow‐up computed tomography angiography was recommended for November 2024, along with regular outpatient follow‐up. A multidisciplinary approach, including psychological support, was emphasized to address the patient′s depressive symptoms and to facilitate a safe, gradual return to physical activity, considered essential for his recovery and long‐term quality of life. Table 1 shows the timeline of the clinical events.

**Table 1:** CARE‐style timeline of the patient′s clinical course.

Date	Clinical event	Diagnostic/therapeutic action	Comment
April 2024	Previous medical history	Electrophysiological study and successful ablation of a left posteroinferior accessory pathway for WPW syndrome	Relevant cardiovascular history
Before September 2024	Baseline status	37‐year‐old male musician with systemic arterial hypertension treated with losartan 50 mg twice daily and amlodipine 5 mg twice daily	Active heavy metal performer
Immediately before symptom onset	Potential precipitating exposure	Repetitive headbanging involving rapid and forceful cervical flexion, extension, and rotation	Potential mechanical trigger
Following exposure	Symptom onset	Severe headache and transient focal neurological symptoms	Symptoms prompted medical evaluation
September 2024	Hospital admission	Admission with ischemic stroke secondary to left ICA dissection	Further neurological and vascular evaluation performed
September 2024	Neurological examination	BP 130/80 mmHg; preserved muscle strength; no focal neurological deficits; NIHSS 0	No measurable neurological deficit at examination
September 2024	Initial vascular imaging	CTA demonstrated a subocclusive thrombotic component involving the distal cervical and intracranial petrous segments of the left ICA, with distal hypoperfusion	Further angiographic assessment performed
September 18, 2024	Definitive vascular assessment	Cerebral angiography confirmed left ICA dissection involving the petrous segment, approximately 5.71 mm in length, with approximately 80% luminal stenosis	Definitive vascular diagnosis documented
September–October 2024	Inpatient treatment	Strict blood pressure control and antithrombotic therapy; dabigatran 150 mg twice daily and pantoprazole 40 mg once daily	Conservative medical management
Hospital discharge	Discharge	Advice to avoid abrupt cervical movements and continue outpatient follow‐up	Professional activities subsequently performed remotely
After discharge	Psychological/functional follow‐up	Fear of resuming physical activity and returning to the gym; depressive symptoms and mood changes	Psychological support emphasized
Two days before outpatient consultation	Psychological treatment	Escitalopram 10 mg initiated	Ongoing psychological/clinical follow‐up
November 2024	Planned vascular follow‐up	CTA scheduled to assess vascular evolution	Imaging result unavailable in current manuscript
Last documented follow‐up	Clinical outcome	Continued outpatient follow‐up and remote professional activity	Final neurological status, medication adherence, recurrent symptoms, adverse events, and imaging outcome require confirmation

*Note:* Source: Table by authors.

Abbreviations: BP, blood pressure; CTA, computed tomography angiography; ICA, internal carotid artery; NIHSS, National Institutes of Health Stroke Scale; WPW, Wolff–Parkinson–White syndrome.

Follow‐up CT angiography was scheduled for November 2024 to assess vascular evolution; however, the imaging report was not available for review at the time of manuscript preparation. At the most recent documented clinical follow‐up, no new ischemic events had occurred, and neurological status remained preserved. Table 2 shows a summary of the case described.

**Table 2:** Clinical case summary table.

Category	Details
Identification	L.S.R.M., 37‐year‐old male, married, musician
Clinical history	Admitted in September 2024 with a diagnosis of ischemic stroke secondary to left internal carotid artery (ICA) dissection, following a possible minor trauma during heavy metal performances involving the practice of headbanging. The patient reports fear of resuming physical exercise and returning to the gym, as well as depressive symptoms and mood changes. Escitalopram 10 mg was initiated 2 days prior to evaluation
Past medical history	Electrophysiological study and successful ablation of a left posteroinferior accessory pathway due to Wolff–Parkinson–White (WPW) syndrome in April 2024. The patient has been working remotely since hospital discharge
Chronic medications	Systemic arterial hypertension under treatment with losartan 50 mg twice daily and amlodipine 5 mg twice daily. Anticoagulation with dabigatran (Pradaxa) 150 mg twice daily. Gastric protection with pantoprazole 40 mg once daily in the morning
Physical examination	Blood pressure: 130/80 mmHg. Preserved muscle strength. No focal neurological deficits
NIHSS score	0 (no evident neurological deficits)
Imaging studies	Cranial CT angiography: Findings suggestive of a subocclusive thrombus in the left ICA involving the distal cervical and intracranial petrous segments, associated with signs of distal hypoperfusion in the corresponding arterial territory. Cerebral angiography (September 18, 2024): Left ICA dissection in the petrous segment, measuring approximately 5.71 mm in length, with significant stenosis estimated at approximately 80% of the arterial lumen
Management and follow‐up	Adjustment of pharmacological therapy, with emphasis on strict blood pressure control and continuation of anticoagulation. The patient was advised to undergo follow‐up CT angiography in November 2024 and to continue scheduled outpatient follow‐up.

*Note:* Source: Table by authors.

### 3.1. Patient Perspective

Following hospital discharge, the patient reported fear of resuming physical activity and returning to the gym because of concern about recurrent neurological or vascular complications. He also experienced depressive symptoms and mood changes during recovery, which prompted psychological support and initiation of escitalopram. His main recovery priorities were to regain confidence in physical activity, maintain his professional activities, and reduce the perceived risk associated with returning to his usual routine.

## 4. Discussion

Diagnostic reasoning and alternative etiologies should be considered when evaluating ischemic stroke associated with cervical artery dissection in a relatively young patient. Cardioembolic disease was clinically relevant given the patient′s history of Wolff–Parkinson–White syndrome; however, he had undergone successful ablation of a left posteroinferior accessory pathway in April 2024. Atherosclerotic disease was considered less likely given the patient′s age and the angiographic pattern of the dissection, although systemic arterial hypertension represented a relevant vascular risk factor. Other potential causes, including systemic vasculitis, connective tissue disorders, migraine‐associated neurological symptoms, and thrombophilia, should be considered in the differential diagnosis when clinically indicated.

Dynamic angiographic assessment during cervical flexion, extension, and rotation was not performed, as it was not considered clinically necessary once the diagnosis had been established. Such studies may offer additional insight into mechanical vascular compression or excessive arterial mobility and could be considered in future investigations of this proposed mechanism.

Management of ICA dissection includes strict blood pressure control and antithrombotic therapy, aiming to prevent lesion progression and recurrent ischemic events [4]. In this case, anticoagulation with dabigatran 150 mg twice daily was selected as the antithrombotic strategy during the acute management of the documented dissection, with the rationale of reducing thromboembolic risk in a markedly stenotic, dissected arterial segment. Evidence comparing anticoagulation with antiplatelet therapy in cervical artery dissection remains mixed [3, 5–7]; a 2024 American Heart Association scientific statement and a recently updated Cochrane systematic review both concluded that neither strategy has demonstrated clear superiority and that treatment should be individualized according to lesion anatomy, ischemic presentation, bleeding risk, and the treating team′s clinical judgment [8, 9]. In the acute phase, intravenous thrombolysis has also been associated with improved functional outcomes in dissection‐related stroke without an increased risk of symptomatic hemorrhage and remains a reasonable option in eligible patients [10]. During treatment, the patient should be monitored for bleeding complications and counseled regarding activities associated with trauma or abrupt cervical movements.

Endovascular treatment, including carotid artery stenting with or without percutaneous transluminal angioplasty (PTA), may be considered in selected patients with persistent hemodynamically significant stenosis, recurrent cerebral ischemia despite optimal antithrombotic therapy, an enlarging pseudoaneurysm, or failure of medical management. Because our patient remained neurologically stable, with preserved distal perfusion and no evidence of recurrent ischemia, conservative treatment with anticoagulation and close imaging surveillance was considered appropriate.

Reports describing cervical artery dissection associated with headbanging remain exceedingly rare. Most published cases involve vertebral artery dissection, with fewer describing ICA involvement. As in previously reported patients, our case involved a young individual without major vascular risk factors who developed neurological symptoms shortly after repetitive, vigorous cervical movements. This growing body of evidence suggests that clinicians should routinely inquire about recent recreational or occupational neck movements when evaluating cryptogenic stroke in young adults.

## 5. Conclusion

This case supports the possibility that repetitive vigorous cervical movements associated with headbanging may act as a precipitating factor for ICA dissection in susceptible individuals. The absence of follow‐up imaging represents a limitation in the management of this case, as it precludes radiographic assessment of vascular healing and long‐term vessel status. Despite the fact that causality cannot be established from a single observation, clinicians should consider this activity when evaluating young patients presenting with cervical artery dissection or ischemic stroke without an obvious cause.

## Funding

No funding was received for this manuscript.

## Conflicts of Interest

The authors declare no conflicts of interest.

## Data Availability

The data that support the findings of this study are available upon request from the corresponding author. The data are not publicly available due to privacy or ethical restrictions.

## References

[1] Debette S. and Leys D. , Cervical-Artery Dissections: Predisposing Factors, Diagnosis, and Outcome, Lancet Neurology. (2009) 8, no. 7, 668–678, 10.1016/S1474-4422(09)70084-5. 19539238

[2] Kirsch M. and Koury R. , Carotid Artery Dissection From Minor Cervical Trauma: The Role of Headbanging in Young Adults, Journal of Stroke & Cerebrovascular Diseases. (2022) 31, no. 4, 106404.

[3] Schievink W. I. , Spontaneous Dissection of the Carotid and Vertebral Arteries, New England Journal of Medicine. (2001) 344, no. 12, 898–906, 10.1056/NEJM200103223441206. 11259724

[4] Naylor A. R. , Ricco J. B. , de Borst G. J. , De Carlo M. , Halliday A. , Kakkos S. K. , Markus H. S. , McCabe D. J. , Sillesen H. , and van den Berg J. C. , Editor′s Choice – European Society for Vascular Surgery (ESVS) 2023 Clinical Practice Guidelines on the Management of Atherosclerotic Carotid and Vertebral Artery Disease, European Journal of Vascular and Endovascular Surgery. (2023) 65, no. 1, 7–111, 10.1016/j.ejvs.2022.04.011. 35598721

[5] Caso V. , Paciaroni M. , and Bogousslavsky J. , Environmental Risk Factors and Cervical Artery Dissection, Frontiers of Neurology and Neuroscience. (2020) 45, 47–54. 10.1159/000088134 17290110

[6] Connolly S. J. , Ezekowitz M. D. , Yusuf S. , Eikelboom J. , Oldgren J. , Parekh A. , Pogue J. , Reilly P. A. , Themeles E. , Varrone J. , Wang S. , Alings M. , Xavier D. , Zhu J. , Diaz R. , Lewis B. S. , Darius H. , Diener H. C. , Joyner C. D. , and Wallentin L. , Dabigatran Versus Warfarin in Patients With Atrial Fibrillation, New England journal of medicine. (2009) 361, no. 12, 1139–1151, 10.1056/NEJMoa0905561. 19717844

[7] Markus H. S. , Hayter E. , Levi C. , Madigan J. , and Norris J. , Antiplatelet Therapy Versus Anticoagulation for Cervical Artery Dissection (CADISS): A Randomised Trial, Lancet Neurology. (2019) 18, no. 11, 793–965. 10.1016/S1474-4422(15)70018-9 25684164

[8] Yaghi S. , Engelter S. , Del Brutto V. J. , Field T. S. , Jadhav A. P. , Kicielinski K. , Madsen T. E. , Mistry E. A. , Salehi Omran S. , Pandey A. , and Raz E. , Treatment and Outcomes of Cervical Artery Dissection in Adults: A Scientific Statement From the American Heart Association, Stroke. (2024) 55, no. 3, e91–e106, 10.1161/STR.0000000000000457, 38299330. 38299330

[9] Avramiotis N. S. , Schaub F. , Thilemann S. , Lyrer P. , and Engelter S. T. , Antithrombotic Drugs for Carotid Artery Dissection: Updated Systematic Review, European Stroke Journal. (2025) 10, no. 2, 339–349, 10.1177/23969873241292278, 39460559. PMC11556626 39460559

[10] Shu L. , Akpokiere F. , Mandel D. M. , Field T. S. , Guerrero C. R. , Henninger N. , Muppa J. , Affan M. , Lodhi O. U. , Heldner M. R. , and Antonenko K. , Intravenous Thrombolysis in Patients With Cervical Artery Dissection: A Secondary Analysis of the STOP-CAD Study, Neurology. (2024) 103, no. 7, e209843. 10.1212/WNL.0000000000209843 PMC11415265 39298709

